# One size does not fit all: notable individual variation in brain activity correlates of antidepressant treatment response

**DOI:** 10.3389/fpsyt.2024.1358018

**Published:** 2024-04-02

**Authors:** Gwen van der Wijk, Yaruuna Enkhbold, Kelsey Cnudde, Matt W. Szostakiwskyj, Pierre Blier, Verner Knott, Natalia Jaworska, Andrea B. Protzner

**Affiliations:** ^1^Department of Psychology, University of Calgary, Calgary, AB, Canada; ^2^Institute of Mental Health Research, Affiliated with the University of Ottawa, Ottawa, ON, Canada; ^3^Department of Cellular & Molecular Medicine, University of Ottawa, Ottawa, ON, Canada; ^4^Hotchkiss Brain Institute, University of Calgary, Calgary, AB, Canada; ^5^Mathison Centre, University of Calgary, Calgary, AB, Canada

**Keywords:** major depression, treatment response, EEG connectivity, EEG complexity, individual variability

## Abstract

**Introduction:**

To date, no robust electroencephalography (EEG) markers of antidepressant treatment response have been identified. Variable findings may arise from the use of group analyses, which neglect individual variation. Using a combination of group and single-participant analyses, we explored individual variability in EEG characteristics of treatment response.

**Methods:**

Resting-state EEG data and Montgomery-Åsberg Depression Rating Scale (MADRS) symptom scores were collected from 43 patients with depression before, at 1 and 12 weeks of pharmacotherapy. Partial least squares (PLS) was used to: 1) identify group differences in EEG connectivity (weighted phase lag index) and complexity (multiscale entropy) between eventual medication responders and non-responders, and 2) determine whether group patterns could be identified in individual patients.

**Results:**

Responders showed decreased alpha and increased beta connectivity, and early, widespread decreases in complexity over treatment. Non-responders showed an opposite connectivity pattern, and later, spatially confined decreases in complexity. Thus, as in previous studies, our group analyses identified significant differences between groups of patients with different treatment outcomes. These group-level EEG characteristics were only identified in ~40-60% of individual patients, as assessed quantitatively by correlating the spatiotemporal brain patterns between groups and individual results, and by independent raters through visualization.

**Discussion:**

Our single-participant analyses suggest that substantial individual variation exists, and needs to be considered when investigating characteristics of antidepressant treatment response for potential clinical applicability.

**Clinical trial registration:**

https://clinicaltrials.gov, identifier NCT00519428.

## 1 Introduction

Neuroscience research on major depression (MD) has greatly improved our understanding of the brain alterations accompanying the disorder. Accumulating studies comparing patients with MD to controls have highlighted both local and global alterations in brain network function, indicating that MD might best be characterized as a network disorder (1, 2). Studies have also examined relationships between brain network characteristics and antidepressant treatment success; this is especially relevant given the variability in treatment outcomes in MD (e.g. 3). Despite high hopes for the application of such research in clinical practice, findings have been variable, and no robust diagnostic or prognostic information for individual patients have been reported to date, or are being implemented routinely (4, 5).

Functional connectivity, which is an index of brain network function that measures the level of synchronized activity between brain regions, has shown promise for revealing network characteristics associated with treatment success (6–9). Electroencephalography (EEG) studies investigating associations between treatment success following pharmacotherapy and functional connectivity found that weaker low frequency (delta, theta and alpha) connectivity at baseline, and a decrease in connectivity at these frequencies in right frontal and temporal electrode pairs was associated with better outcomes (6, 9). However, increased alpha connectivity with treatment has also been associated with better treatment outcomes (8). In the beta frequency band, some studies found lower pre-treatment connectivity and an early increase in connectivity, again mostly at frontal, temporal and central sites, to be associated with a better response (7, 8), while others did not find any treatment-related effects in beta connectivity (6), further highlighting the variability of findings in this context.

Researchers have also investigated network dynamics in MD by examining complexity in brain signals, which provides complementary information to more traditional measures of brain network function (10). Signals are considered to be complex when they have both stochastic and deterministic properties, and thus are neither completely predictable nor entirely random (11). Some studies suggest that patients with MD exhibit greater signal complexity than controls (12–15), and decreases in complexity have been associated with symptom improvement (16, 17). In contrast, Čukić and colleagues (18) found higher complexity in patients in remission from MD compared to both currently depressed patients and controls. Importantly, most of the discussed studies assessed complexity only at high temporal resolutions (1-10 milliseconds between datapoints). Our group found no association between treatment response and complexity at these fine temporal scales prior to treatment, but demonstrated that greater treatment response was associated with greater complexity at lower temporal resolutions (i.e. 20-40ms; 19).

These variable findings have been attributed to the intrinsically heterogeneous nature of depression (3, 20, 21), and not just in terms of symptom profiles, which alone can present with over 1000 unique symptom profiles (22). Other sources of heterogeneity include age of onset, chronicity and severity of depression, psychiatric comorbidities, as well as sex and gender differences (23, 24). In this context, group analyses using small to moderate samples could easily lead to variable findings, which may account for difficulties identifying robust diagnostic or prognostic classifiers. Group analyses tend to capture central tendencies in the data and treat individual variation outside of these common features as noise; as such, this might result in somewhat different commonalities depending on the patient sample included in each study. Evidence of such individual divergence from group level findings in brain recordings was recently shown by our group in MD, where we quantified individual differences in fMRI functional connectivity (resting state and task, with task effects revisualized) from patients and controls. Individual differences in functional connectomes accounted for >40% of the explained variance in the data. Group differences were significant but much smaller for sex, depression diagnosis and its treatment which accounted for only about 5% of the variance together (25, preprint). These results underscore the importance of exploring individual variation in relation to group findings in psychiatric research.

Based on this work, we sought to characterise the extent to which group level findings are able to describe individuals. We examined EEG connectivity and complexity in data from a registered clinical trial comparing mono- vs. dual-therapy with antidepressant medications (escitalopram and bupropion) whose efficacy has already been established, and for which group results on brain function have been investigated and reported (e.g., 19, 26–28). In our study, we novelly focused on brain comparisons between MD groups with different treatment outcomes and examined how well such group differences translate to individuals. To our knowledge, the latter has not yet been done in the context of MD.

Similar to other studies in this field (e.g., 7, 8, 18), our sample consisted of 43 well-characterized patients, receiving one of three antidepressant medication regimens (escitalopram, buproprion or buprioion+escitalopram) for 12 weeks. For group analyses, patients were divided into eventual antidepressant pharmacotherapy responders (≥50% symptom improvement on the Montgomery-Åsberg Depression Rating Scale [MADRS] from baseline to 12 weeks of treatment) and non-responders (<50% improvement). EEG was measured at baseline, and after 1 and 12 weeks of treatment. We identified patterns of change in EEG connectivity and complexity at the group level in responders and non-responders, and examined the extent to which each individual’s results conformed to their own group’s pattern through single-participant analyses. Based on the most consistently reported findings from previous literature, we expected that as a group, responders would exhibit decreased connectivity at lower frequencies (delta, theta and alpha) with treatment, and decreased complexity in response to pharmacotherapy (6, 7, 9, 16). Based on our work quantifying individual differences in MD with fMRI (25, preprint), we expected significant individual variation around the patterns found in responder and non-responder groups. Here we map what this variation looks like.

## 2 Methods

**Figure 1:**
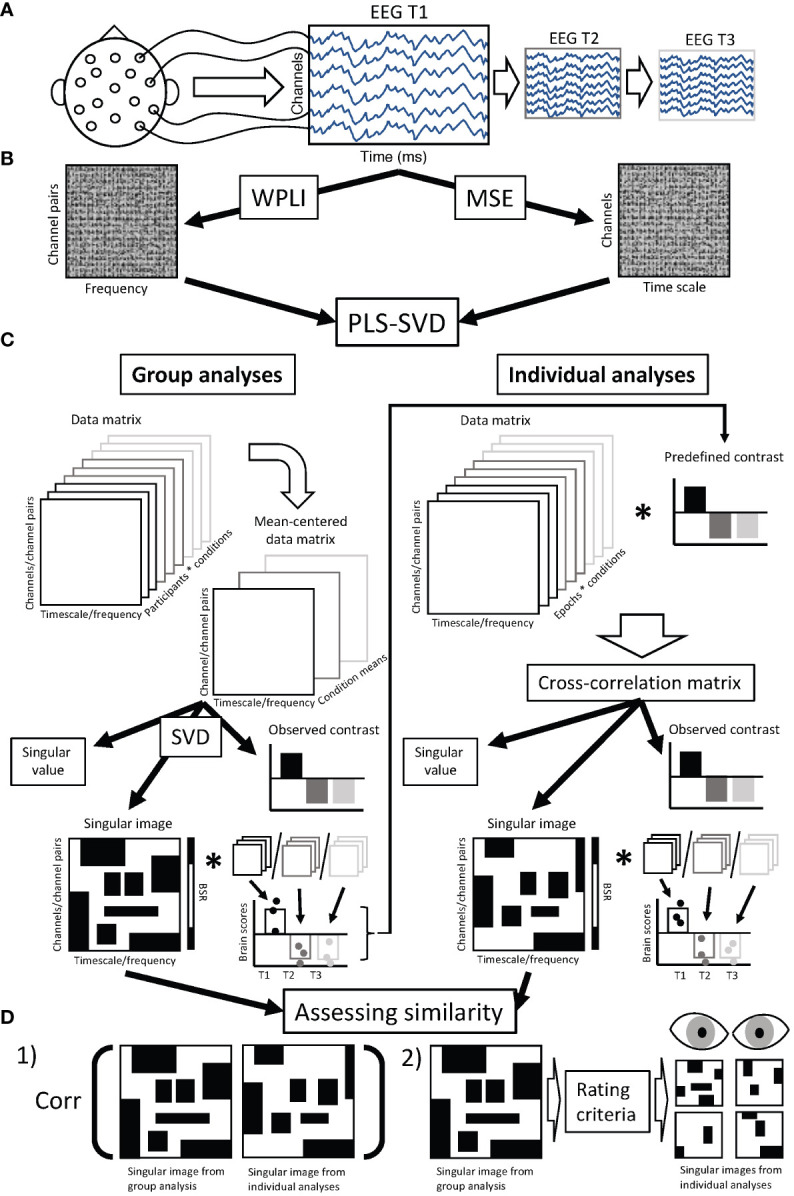
Chart diagram of data analysis procedure. **(A)** Resting state EEG was collected from each participant at three time points: prior to treatment (T1), 1 week after starting treatment (T2), and 12 weeks after starting treatment (T3). **(B)** Preprocessed EEG data was used to calculate connectivity (weighted phase lag index - WPLI) and complexity (multiscale entropy - MSE) for each participant and assessment session, resulting in a data matrix of dimensions 99 (frequencies) * 378 (channel pairs) for WPLI and 28 (channels) * 20 (timescales) for MSE, for each individual and assessment session. **(C)** These data matrices were aggregated and entered into PLS-SVD analyses. For the group analyses, we used regular (mean-centering) PLS. In such analyses, the entered data matrix (containing WPLI/MSE values averaged over epochs for each participant) is mean-centered, meaning the normalized average is calculated within each condition (in our case assessment sessions). This creates a mean-centered data matrix, which is subjected to singular value decomposition (SVD). This results in latent variables (LVs), the first accounting for most of the variance, and each remaining LV accounting for additional parts of the remaining variance (only one LV is illustrated). Each LV consists of a singular value, an observed contrast and a singular image. The singular value indicates the strength of the effect revealed by the LV, and is used to assess the significance of the LV through permutation testing. The observed contrast reveals the condition differences the LV represents. The singular image highlights the elements (in our case, timescales/frequencies and channels/channel pairs) where differences in connectivity/complexity are identified. A bootstrapping procedure is used to identify the elements that show stable differences across participants (indicated by bootstrap ratios). The singular image is multiplied by the original data matrices for each participant and session to calculate brain scores, which reveal the individual variation in the expression of the condition contrast. Brain scores are used to determine the 95% confidence interval around the condition means, and can therefore show the reliability of the observed contrast. The reliable observed contrasts from the group analyses were used in the individual analyses, for which we employed non-rotated PLS. In these analyses, the data matrices (WPLI/MSE values from single epochs), were multiplied with the condition contrasts observed in the group analyses to examine if the same contrasts were present in individual participants. The same components (singular value, observed contrast and singular image) are then extracted from the cross-correlation matrix and assessed the same way (through permutation testing and bootstrap resampling). **(D)** We assessed the similarity between group and individual findings in two ways. 1) We correlated the singular image from the group analysis with the singular images from each individual’s analysis. 2) We used the singular image from the group analysis to determine the main features of the group findings, which were then used as rating criteria by two independent, blind raters using visual inspection to rate whether or not the singular images from individual analyses matched the singular images of the group findings. While the assessment of similarity was focused on the spatiotemporal pattern (singular images), significance and the match of observed contrasts was also checked before assessing similarity of singular images. EEG, electroencephalography; T1, assessment session 1 (prior to treatment); T2, assessment session 2 (at 1 week of treatment); T3, assessment session 3 (at 12 weeks of treatment); WPLI, weighted phase lag index; MSE, multiscale entropy; PLS, partial least squares; SVD, singular value decomposition; BSR, bootstrap ratio; corr, correlation.

An overview of the data collection, processing, and analysis procedure can be found in **Figure 1**.

### 2.1 Participants

Fifty-three adults with a primary diagnosis of major depressive disorder (MD), as assessed by a psychiatrist with the Structured Clinical Interview for the Diagnostic and Statistical Manual of Mental Disorders-Fourth Edition (Text Revision) DSM-IV-TR [SCID-IV-TR] (29), participated in this study, as previously described (19). Briefly, patients were excluded if they had any other Axis I disorder (except for anxiety disorders), recent (< 6 months ago) problems with substance abuse/dependence, an unstable medical condition, significant suicide risk, seizure history, or if they had been previously treated for their current depressive episode with the study medications. Medicated patients underwent a supervised washout period prior to study commencement (>5 weeks for fluoxetine, 1 week for other medications, consistent with wash-out protocols). As part of a clinical trial conducted between August 2007 and March 2012 (28, 30), patients received either escitalopram (ESC) and placebo, bupropion (BUP) and placebo, or a combination of the two medications for 12 weeks. Assignment to a specific treatment regimen was randomized (double blind).

Depressive symptoms were assessed using the MADRS (31, 32), every week during the first 4 weeks, and biweekly for the remaining 8 weeks. Dosage was increased if tolerated and remission was not yet reached (average dose at 12 weeks for the current sample: dual treatment: ESC = 32 mg, BUP = 379 mg; monotherapy: ESC = 34 mg, BUP = 425 mg). All patients had a baseline MADRS score ≥ 22. Patients whose MADRS scores improved ≥50% from baseline to 12 weeks were considered responders (R), while those who improved <50% were considered non-responders (NR). Due to participant drop-out and issues with EEG data quality, 10 participants were excluded, leaving 43 participants for data analysis (i.e. complete datasets at baseline, week 1 and 12). Of these, 25 were responders and 18 were non-responders. Demographic and clinical characteristics can be found in **Table 1** (statistically compared on pertinent variables). All participants provided written informed consent and were reimbursed $30 CAD/testing session. This study was approved by the Royal Ottawa Health Care Group and University of Ottawa Research Ethics Boards.

**Table 1:** Demographic and clinical characteristics (means ± standard error) of antidepressant treatment responders and non-responders.

	Responders (N = 25)	Non-responders (N = 18)	Statistics
*Sex (F/M)*	14/11	9/9	χ^2^(3) = 0.15, *p* = .70
*Age*	35.1 ± 2.1 (range: 19-57)	44.8 ± 2.7 (range: 20-63)	t(35) = 2.77, *p* = .009*
*Education (years)*	15.4 ± 0.5	16.3 ± 0.6	t(34) = 1.20, *p* = .24
*Race/Ethnicity*	3 Asian; 22 White	1 African; 17 White	*p* = .48 (Fisher’s exact test)
*Comorbid anxiety (Yes/No)*	3/22	3/15	*p* = .68 (Fisher’s exact test)
*Treatment regimen (ESC+BUP/BUP+placebo/ESC+placebo)*	12/6/7	5/6/7	χ^2^(5) = 1.79, *p* = .41
*Baseline MADRS score*	29.4 ± 0.9	32.2 ± 1.1	t(36) = 1.91, *p* = .064
*MADRS score at 1 week*	23.1 ± 1.6	27.9 ± 1.9	t(37) = 1.92, *p* = .063
*MADRS score at 12 weeks*	6.0 ± 1.0	24.9 ± 1.9	t(26) = 8.85, *p* <.001*

Group differences were examined using independent samples t-tests in Excel, unless reported otherwise. * Significance at p <.05. F, female; M, male; ESC, escitalopram; BUP, bupropion; MADRS, Montgomery-Åsberg Depression Rating Scale.

We conducted power analyses in G*Power (33) to assess the statistical power our sample afforded us. As no established way exists to conduct power analyses for our multivariate statistical approach (partial least squares - see below), we estimated power based on the univariate model that most closely matched our experimental design, namely a 2 (responders vs non-responders) x 3 (baseline, week 1, week 12) mixed ANOVA. We determined that our sample size is sufficient to detect small within-subject and moderate between-subject effects at >80% power, and these calculations are in line with sample size guidelines proposed by Cohen and colleagues (34).

### 2.2 EEG data collection

Resting state EEG recordings were collected before the start of treatment (baseline), 1 week and 12 weeks after treatment initiation at the Royal Ottawa Mental Health Centre. Participants abstained from caffeine and nicotine >3 hours prior to testing, and did not take any psychotropic medications, other than the prescribed antidepressants. Two 3-minute resting-state EEG recordings were collected, one with eyes open (EO) and one with eyes closed (EC), while participants sat in a temperature- and light-controlled testing chamber. Ip and colleagues (35) showed that 3-minute EEG recordings are enough to extract reliable EEG characteristics in the theta, alpha and beta bands using a test-retest design. Gudmundsson and colleagues (36) furthermore, examined reliability in EEG spectral power, complexity and connectivity, and found decent reliability with 40 seconds of data included in analysis. The order of EO and EC testing was counterbalanced between participants and sessions. The rationale for these short eyes open/eyes closed recordings was to mitigate the possibility of sleepiness contaminating the data. Furthermore, most data collection was done during the day (i.e., not first thing in the morning or in the evening), though variability existed. EEG was recorded using 32 Ag/AgCl (silver chloride) electrodes embedded in a cap (EasyCap, Inning am Ammersee, Germany), with electrodes positioned according to a variant of the 10-20 system (37). AFz served as the ground, and the average of the two mastoid channels (Tp9/Tp10) was used as the reference. Four additional channels were placed outside the left and right eye canthi, and above and below one eye, to monitor electrooculographic (EOG) activity. Data was sampled at 500 Hz, and impedance was <5KΩ (BrainVision Recorder, Gilching, Germany).

### 2.3 EEG preprocessing

EEG data were preprocessed using EEGLAB v13.4.4b (38) in MATLAB 2014 (The MathWorks, Inc., Natick, Massachusetts). Raw EEG data were bandpass filtered (0.5-55 Hz; slope: 12 dB/octave) using ERPlab’s IIR butterworth filter, notch filtered at 60 Hz (lower and upper edge: 55-65 Hz) using EEGlab’s basic FIR filter and segmented into 2s epochs. We used an independent component analysis (ICA) to identify and eliminate noise and ocular artifacts. Channels with excessive noise or drift were excluded from the ICA procedure, and subsequently interpolated using EEGlab’s spherical spline interpolation function. The average number of interpolated channels was 0.11, and no more than two channels were interpolated for each participant and session (34). Epochs were visually inspected following ICA, and those with remaining artifacts were manually rejected. An average of 85.7 (range: 63-113) artifact free epochs were obtained per participant, state (EC/EO) and session (baseline, week 1 & 12), including 28 electrodes (Fp1/2; F3/4; F7/8; FC1/2; FC5/6; C3/4; CP1/2; CP5/6; P3/4; P7/8; T7/8; O1/2; Fz/Cz/Pz/Oz). The two reference channels and two additional channels that provided low data quality for most participants (FT9/FT10) were excluded from analysis. There was no statistical difference between responders and non-responders in number of artifact-free epochs per session or state (*p-*values:.09-.9).

### 2.4 Connectivity analysis

Functional connectivity, as quantified by the weighted phase lag index (WPLI), was calculated for each unique combination of the 28 channels (378 pairs) using the open source Fieldtrip toolbox (39) in MATLAB. WPLI is a modified version of the phase lag index (PLI), which was first described in 2007 by Stam and colleagues (40). It estimates connectivity by calculating the phase angle difference between EEG signals from two channels for each time point, and determining the consistency in these phase lags over time. As such, if the difference in phase between two channels is similar over time, the PLI will be high, indicating high connectivity between two channels. An advantage of the PLI compared to other EEG connectivity measures is that it is less sensitive to volume conduction, because it disregards any phase lags of 0 and π. The WPLI also takes into account that phase lags can easily turn into leads and vice versa (e.g. a slightly positive phase angle difference can turn into a slightly negative phase angle difference). While the PLI is sensitive to such small disturbances in phase lags, the WPLI resolves this issue by giving greater weight to angle differences around 0.5π and 1.5π (41). The result is a value between 0 and 1, with higher values indicating stronger connectivity. WPLI was calculated in two ways, first across epochs, as is commonly used and enables direct comparisions with previous findings, and then within epochs, which is less commonly used, but enables single-participant analyses.

#### 2.4.1 Across-epoch WPLI

Phase information was first extracted for each epoch, channel and frequency bin (0.5-50 Hz, 0.5 Hz bins) using Fieldtrip’s fast Fourier transformation algorithm. A Hanning taper was used for the lower frequencies (0.5-30 Hz), while a multi-taper using the discrete prolate spheroidal sequences (dpss) method with 2 Hz smoothing was applied to the higher frequencies (31-50 Hz), to optimize sensitivity of spectral content at each frequency. WPLI values were then calculated by considering the consistency of phase lags over epochs at each frequency bin for all channel pairs using Fieldtrip’s connectivity function. Across-epoch WPLI was used for the group analyses. This across-epoch method is not suitable for single-participant analyses, because it does not allow calculation of WPLI for individual epochs. Therefore, a second approach was used to calculate WPLI for the single-participant analyses (34).

#### 2.4.2 Single-epoch WPLI

Instead of extracting one phase value per epoch, phase was determined for each time point within an epoch using a time-frequency transformation with Morlet wavelets in the time domain. To have reasonable temporal and frequency resolution, the length of the wavelets was increased with frequency in regular steps, from 3 cycles at 4 Hz to 7 cycles at 50 Hz (34). Frequencies below 4 Hz were not included as the length of our epochs (2 seconds) was too short to provide reliable estimations at these frequencies (i.e. 3 cycles of a 1 Hz wavelet are longer than 2 seconds). WPLI could then be calculated for individual epochs by examining the consistency of phase lags over time points within each epoch (34). These individual epoch data were used for the individual connectivity analyses. To confirm that this single-epoch approach provides similar results at the group level as the across-epoch approach, we also averaged these data over epochs for each participant and ran the same group level analyses.

### 2.5 Brain signal complexity analysis

Multiscale entropy (MSE) was used to quantify brain signal complexity. An advantage of MSE over other measures of complexity is that it incorporates multiple time scales. This feature is important because it differentiates between signals that are purely random (e.g., white noise) and those comprised of both random and deterministic components (e.g., 1/f or coloured noise). Signals that are purely random show a rapid decline in the MSE curve with increasing scale whereas those with temporal inter-dependencies will have a more gradual shift in the MSE curve (42, 43). A detailed description and theoretic background for MSE is outlined in Costa and colleagues (11). In short, MSE estimates the regularity of a signal by evaluating the ratio of similar patterns of different lengths repeating over several time scales. It is calculated in two steps. First, the raw signal is resampled several times to create data sequences that represent different temporal scales. Essentially, an increasing number of non-overlapping data points are averaged into one new data point. The first timescale is the (cleaned) raw time series. With a sample rate of 500Hz in the current study, time scale 1 had a temporal resolution of 2 milliseconds between data points. For time scale 2, two consecutive data points were averaged, yielding a temporal resolution of 4 milliseconds; for time scale 3, averaging occurs over three time points yielding a temporal resolution of 6 milliseconds, and so on. The coarsest scale used in this study was 20 (temporal resolution of 40 milliseconds), to ensure a sufficient number of data points (minimum 50) for the sample entropy calculation.

Next, sample entropy is calculated at each time scale. Sample entropy determines the natural logarithm of the ratio of patterns of length *m* over patterns of length *m+1* repeated within one epoch. This gives a value between 0 and 1, with higher numbers indicating a less predictable/more variable signal (i.e. fewer patterns of length *m+1* compared to the number of patterns of length *m*). In line with previous studies, (e.g. 19, 44) and guidelines outlined by Richman and Moorman (45), we set parameter *m* to 2, while the similarity criterion *r*, which determines which points in the time series are considered to be ‘the same’, was set to 0.5 (i.e. two data points were treated as indistinguishable if their amplitudes differed <50% of the standard deviation of the time series). MSE was calculated for each epoch and electrode at each time scale, using the algorithm available at www.physionet.org/physiotools/mse/. Single epoch MSE data were used for statistical analyses at the individual level. MSE values were also averaged over epochs to provide one MSE value for each electrode and time scale per participant, session (baseline, week 1 and 12) and state (EC and EO), which were used for group level analyses.

### 2.6 Regression of age effects

To control for differences in age between responder and non-responder groups (see **Table 1**), and because both brain signal complexity and connectivity have been observed to change with age (19, 46–49), age was regressed out of the data before the statistical group comparisons using an in-house MATLAB script (50, 51).

### 2.7 State contrasts

Consistent with MSE and connectivity differences between EO and EC states observed in previous studies (19, 52–55), we found strong EO/EC effects in our analyses that masked changes occurring over assessment sessions (see **Supplementary Figures S1**, **S2**). Therefore, we performed analyses on EO and EC data separately. To maximize the chance of replicating group findings at the individual level, we performed single-participant analyses on the data showing the strongest effects (EC for WPLI, EO for MSE), and present those group findings below (other group findings are presented in **Supplementary Materials** [**Figures S3**, **S4**]).

### 2.8 Statistical analyses with PLS-SVD

Partial least squares with singular value decomposition (PLS-SVD) is a multivariate statistical approach that can detect condition- and/or group-related differences in whole-brain variables (56, 57). Briefly, PLS-SVD calculates the normalized average across participants within each condition (in this case, responder status and assessment sessions) for each element in the brain characteristic matrices (in this case, frequencies and electrode pairs for WPLI, or time scales and electrodes for MSE). Then, this mean-centered matrix is decomposed using SVD into orthogonal latent variables (LVs) that account for most of the covariance between groups/conditions and brain characteristics, revealing the optimal associations between specific groups/conditions and spatiotemporal patterns in the brain. LVs contain several components. One is the singular value, which indicates the strength of the effect the LV represents. Another component holds the condition contrast, which reveals the linear combination of weights capturing differences between conditions (groups and assessment sessions in our data). The third component contains the element loadings (singular image), which represent the pattern of the specific data elements (in this study frequencies and electrode pairs for WPLI, and time scales and electrodes for MSE) that show the given contrast. These element loadings are used to compute brain scores: the dot product of the element loadings with each participants’ data for each assessment session. Brain scores represent the extent to which each participant expresses the given contrast in a single number per participant and condition (see **Figure 1C**).

Statistical testing occurs at two levels in PLS-SVD analyses. First, the overall significance of the LV is determined using permutation tests. In each permutation, the data are randomly shuffled between conditions (within participants) and between groups, and PLS-SVD analysis is performed on the shuffled data just as on the actual data. LVs are considered significant when their singular value is more extreme than 95% of the singular values calculated from the randomly shuffled data (corresponding to *p* <.05). In the current study, 500 permutations were performed for each analysis. Second, the stability of the identified pattern across participants is established through bootstrap resampling. In essence, the PLS-SVD analysis is repeated with different subsamples of participants, to see how consistently each electrode pair/electrode and frequency/time scale display the identified pattern of differences across the whole sample. This consistency is quantified as a bootstrap ratio (BSR), which is calculated by dividing the element loadings by the standard error of the created bootstrap distribution for each element. In addition to determining the stability of the pattern, bootstrap resampling also protects against the influence of outliers, as subsamples with and without the outlier would produce different outcomes, thereby decreasing the consistency of the findings (i.e. the bootstrap ratio). In practice, this means that effects that are driven largely by an outlier get attenuated. Bootstrap ratios are similar to z-scores, with absolute values ≥ 3.1 corresponding to ~99% confidence interval. In this study, bootstrap resampling was performed 200 times. As each statistical test is computed in one mathematical step, no correction for multiple comparisons is necessary (56). P-values indicating significance levels, and percentage of crossblock covariance explained (PCCE) are reported for each LV of interest. PLS-SVD analyses were applied both at a group and individual level.

### 2.9 Group level analyses

Both groups (responders vs. non-responders) and all sessions (baseline, 1 & 12 weeks of treatment) were entered in four PLS-SVD analyses: two for connectivity (EO/EC states separately) and two for complexity (EO/EC). As all showed interaction effects between groups and assessment sessions, two additional analyses were run for each analysis for responders and non-responders separately, again including all sessions. The p-values of these follow-up analyses were corrected for multiple comparisons using the Bonferroni method.

The input data consisted of across-epoch WPLI/averaged MSE values, organized into 2D matrices with n * k rows, and m * t columns, with n being the number of participants (R: 25; NR:18) and k the number of conditions (assessment sessions: 3). M and t represent the spatiotemporal elements, namely the number of electrode pairs (378) and frequencies (99) for the WPLI analyses and the number of electrodes (28) and timescales (20) for the MSE analyses. The same procedure was followed for the averaged, single-epoch WPLI data (*Methods – Connectivity analyses*).

### 2.10 Single-participant analyses

Non-rotated (hypothesis-driven) PLS analyses were performed for each individual, using single-epoch EC WPLI data, and single-epoch EO MSE data. Non-rotated PLS was chosen because it allows one to determine whether and to what extent a specific, predefined contrast is present in the data (57). Using the contrasts (patterns of differences in connectivity and complexity between groups and across sessions) found in the group analyses, non-rotated PLS was used to test whether each individual followed the pattern of change observed in responder and non-responder groups. Single epoch WPLI/MSE values were organized into similar 2D matrices as described for the group analyses, only now each participant had their own matrix, with the n dimension representing the number of epochs instead of participants (57). No correction for multiple comparisons was applied, as these analyses aimed to replicate group findings in separate datasets for each individual.

The similarity of the individual PLS outcomes to the group PLS-SVD outcomes was quantified in two independent ways to balance the advantages and suitability of quantitative and qualitative measures (see **Figure 1D**). First, the similarity was estimated quantitatively, by correlating the stable (|BSR| > 2, corresponding to ~95% confidence interval) element loadings (i.e. the spatiotemporal brain pattern) of the group results with the element loadings of each participants’ individual analysis in MATLAB. For connectivity, the element loadings from the group analyses on averaged single-epoch WPLI were used for this correlation procedure (Methods – Connectivity analyses). For participants whose PLS analysis was non-significant (*p* >.05) or did not match the predefined contrast (responder/non-responder), indicating a different timing and/or direction of the change highlighted by element loadings, the element loadings were not correlated with the group results and were included as ‘showing no correlation with the group pattern’ in the summaries. The percentage of participants showing moderate-strong correlations (r≥.4; 58) with their own group outcome was considered as a quantitative indicator of how well group results translated to individuals. Second, to balance arbitrary cut-offs, significant individual outcome patterns were visualized and classified by two independent raters, blind to response status, as being similar to either or both the responder or non-responder group patterns, or neither. Important responder and non-responder features were selected based on visual inspection of the most consistent changes across time (i.e. those with |BSR| > 3.1) in the group analyses, and used to construct the rating criteria that both independent raters used to determine similarity. The percentage of participants being classified as conforming to their own group pattern exclusively was determined as a qualitative indicator of the replicability of the group patterns at the individual level.

## 3 Results

### 3.1 Participants

By design, responders had lower MADRS scores at week 12, but not at baseline or week 1 (**Table 1**). Apart from responders being younger than non-responders, the two groups did not differ statistically in clinical and demographic characteristics (**Table 1**). We accounted for the age difference by regressing age effects out of our data before running the statistical tests at the group level.

### 3.2 Group analyses - WPLI

**Figure 2:**
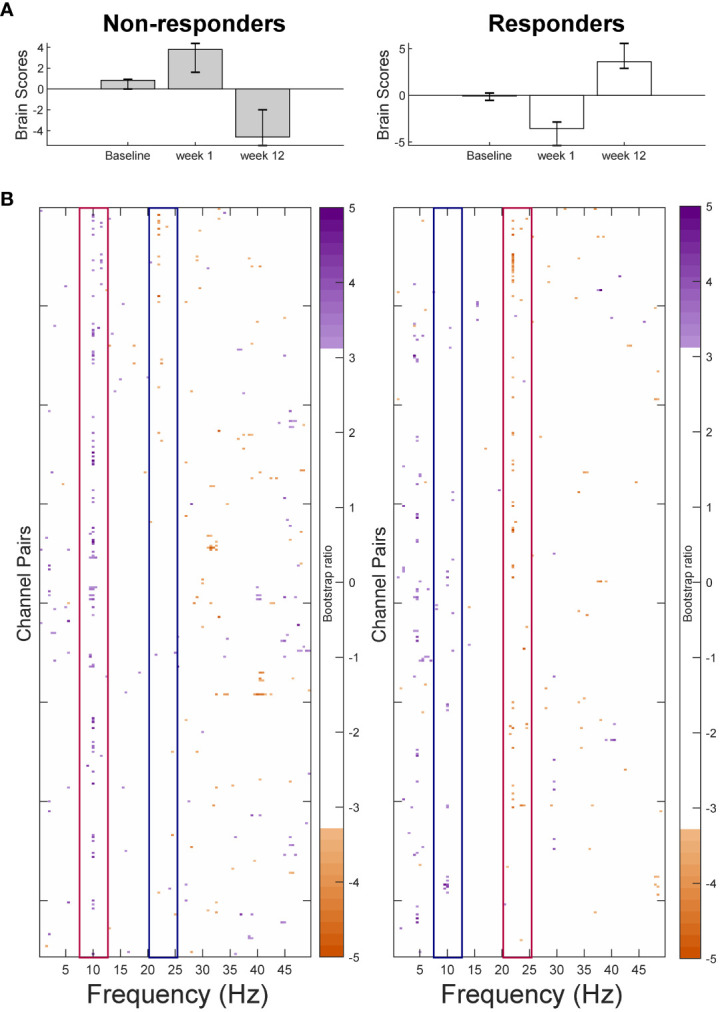
Results from the partial-least squares singular value decomposition (PLS-SVD) analyses examining change in connectivity as measured by weighted phase lag index (WPLI) over the course of antidepressant medication treatment in eventual non-responders (left) and responders (right). Bar graphs **(A)** depict the contrast between assessment sessions within groups, that was significantly expressed across each data set as determined by permutation testing. The statistical image plots **(B)** present the bootstrap ratio maps over all channel pairs (rows) and frequencies (columns). The orange and purple pixels display where the contrast represented by the bar graphs was most reliable across participants as determined by bootstrapping. Positive values (purple) indicate increased WPLI in responders, and decreased WPLI in non-responders from 1 to 12 weeks of treatment, while negative values (orange) indicate decreased WPLI in responders and increased WPLI in non-responders from weeks 1 to 12. To aid interpretability, the most prominent increases in WPLI are highlighted by red boxes, while decreases in WPLI are outlined by blue boxes. As highlighted by these boxes, non-responders showed an increase in alpha and a decrease in beta connectivity from week 1 to week 12 of treatment, while responders showed the opposite pattern.

**Figure 3:**
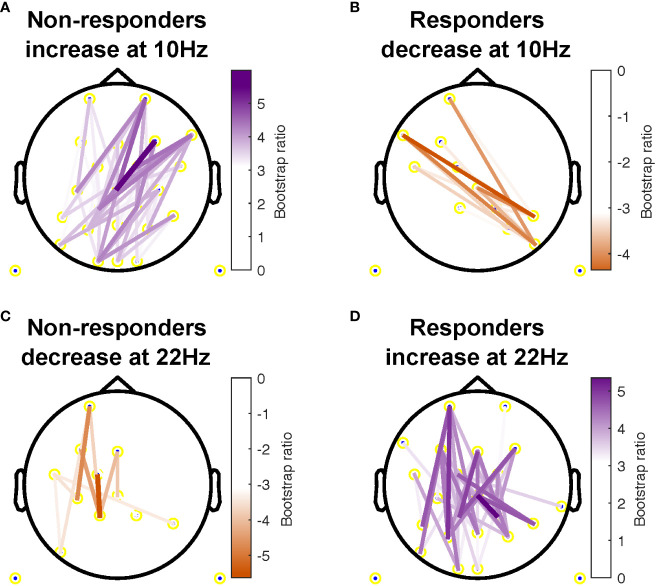
Topographical location of channel pairs showing the most consistent change in connectivity as measured by weighted phase lag index (WPLI) over assessment sessions in non-responders **(A, C)** and responders **(B, D)** at 10Hz **(A, B)** and 22Hz **(C, D)**. Positive values (purple) indicate increased WPLI from 1 to 12 weeks of treatment, while negative values (orange) indicate decreased WPLI from weeks 1 to 12.

The PLS-SVD analysis including both groups and all sessions identified one significant LV (*p* <.001, PCCE = 35.07%). As this LV presented an interaction effect between groups and sessions, two additional analyses for each group separately were run, with the statistical significance threshold corrected to α <.025. These analyses revealed a complex, opposite pattern of change from weeks 1 to 12 in responders (*p*=.024, PCCE=55.8%), but only approached significance in non-responders after Bonferroni correction (*p* = .032, PCCE=56.6%; **Figure 2**). The most prominent frequencies for each group are highlighted by red boxes in **Figure 2**: Non-responders showed a widespread increase in alpha connectivity from weeks 1 to 12 (10Hz), while responders exhibited an extensive increase in beta connectivity (22Hz). Considering the same frequencies in the opposite groups (e.g. alpha in responders; highlighted by blue boxes) revealed more spatially contained changes in the opposite direction: Responders showed a decrease in connectivity at 10Hz, while non-responders showed a decrease at 22Hz. In both groups, changes in alpha connectivity were most pronounced at interhemispheric frontal-to-occipito-parietal electrode pairs but involved.

additional electrode pairs in non-responders. The most consistent beta changes occurred in left intra-hemispheric connections in both groups, but also included right central and parietal channel pairs in responders (**Figure 3**).

The group PLS-SVD analyses performed on the averaged single-epoch WPLI data showed a similar pattern of change in connectivity in responder and non-responders (**Supplementary Material** and **Figure S5**). However, the results spread over multiple frequencies (e.g. from 8-14 Hz instead of dominantly at 10 Hz), which is unsurprising, considering the reduced spectral resolution associated with sliding window approaches (34).

### 3.3 Group analyses - MSE

**Figure 4:**
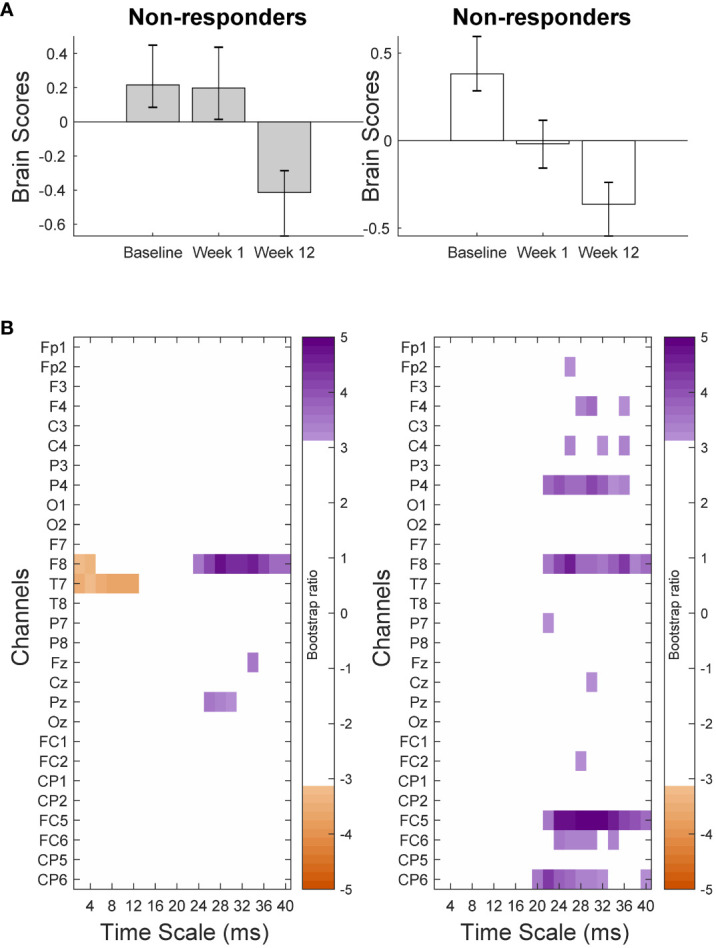
Results from the partial-least squares singular value decomposition (PLS-SVD) analyses examining change in complexity as measured by multiscale entropy (MSE) over the course of antidepressant medication treatment in non-responders (left) and responders (right). Bar graphs **(A)** depict the contrast between assessment sessions within groups, that was significantly expressed across each data set as determined by permutation testing. The statistical image plots **(B)** present bootstrap ratio maps over all channels (rows) and time scales (columns). The colored values display where the contrast represented by the bar graphs was most consistent across participants as determined by bootstrapping. Positive values (purple) indicate decreased MSE, while negative values (orange) indicate increased MSE at week 12 compared to baseline and week 1 in non-responders, and at week 12 compared to baseline in responders.

**Figure 5:**
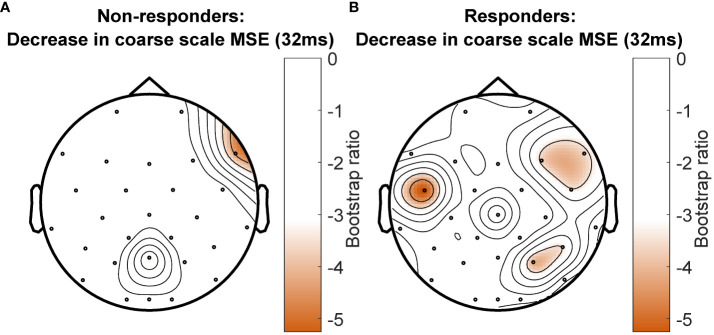
Topographical location of channels showing the most consistent change in complexity as measured by multiscale entropy (MSE) over assessment sessions in non-responders **(A)** and responders **(B)** at a time scale of 32ms between data points. Negative values (orange) indicate decreased MSE at week 12 compared to baseline and week 1 in non-responders, and decreased MSE at week 12 compared with baseline in responders. There was no increase in MSE for any channel at this time scale.

The PLS-SVD analysis examining changes in MSE over time in responders and non-responders identified one significant LV (*p* <.001, PCCE = 82.71%), which revealed an interaction effect. The analyses exploring changes for each group separately each found one significant LV (responders: *p* = .006, PCCE = 93.85%, non-responders: *p* = .02, PCCE = 86.9%, significant at α <.025). Both groups showed a decrease in coarse scale complexity from baseline to 12 weeks, but the timing and extent of change differed. Responders showed an early (starting at week 1) and widespread decrease in coarse scale complexity, while non-responders showed a later (only present at week 12) decrease in coarse scale complexity in limited electrodes (**Figures 4** & **5**). Additionally, fine scale complexity increased only in non-responders.

### 3.4 Individual analyses - WPLI

The pattern of change across assessments identified by the group PLS-SVD examining connectivity was similar regardless of the approach used to calculate WPLI. Specifically, it consisted of a change in connectivity from week 1 to week 12 in both non-responders and responders (**Figure 2**; **Supplementary Figure S5**). Therefore, we applied this contrast in the non-rotated single-participant PLS-SVD analyses (**Figure 1C**). As the non-responder and responder patterns only differed in the direction of change (i.e., increase or decrease in WPLI), only one contrast was defined for each analysis (0 1 -1). This contrast examines changes in WPLI from week 1 to week 12 but leaves the direction of change and at which frequencies this occurs to be determined by the data. All non-responders and 22/25 responders exhibited the predefined pattern of change at an uncorrected significance level (all *p* <.05), of whom 33 (19R/14NR) survived Bonferroni correction (*p* <.001).

**Figure 6:**
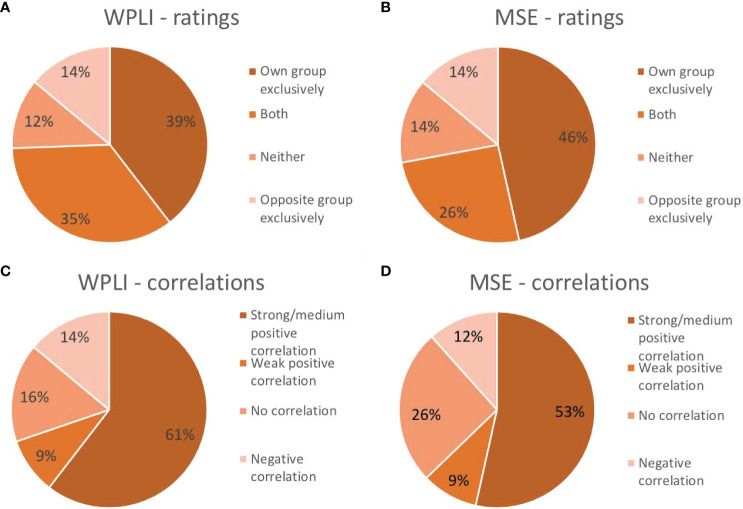
Proportion of participants that exhibited the same pattern as their own group exclusively, the pattern of the opposite group exclusively, both group patterns or neither group pattern in their individual analysis of connectivity as measured by weighted phase lag index (WPLI; **A**) and complexity as measured by multiscale entropy (MSE; **B**). Proportion of participants showing a strong/medium positive correlation (r≥.4), a weak positive correlation (.1<r<.4), a negligible or lack of correlation (-.1<r<.1) or a negative correlation (r<-.1) between their individual outcome matrix and that of their own group for WPLI **(C)** and MSE **(D)**.

The similarity of the pattern of connectivity across channel pairs and frequencies between the individual and group-level results was examined in two ways (see section 2.10 and **Figure 1D** for details). First, each individual’s connectivity pattern was correlated with the connectivity pattern identified in the group analyses. This procedure showed that 60.5% of individual patients exhibited moderate-strong positive correlations (i.e., *r* ≥ .4; 58) between their individual and group outcomes. Another 9.3% showed weak positive correlations (i.e., .1 < *r* < .4), while 14.0% revealed negative correlations between individual and group PLS outcomes. The remaining 16.3% of patients’ individual analyses either correlated negligibly (-.1 < *r* < .1) or did not reach significance and were therefore not correlated (**Figure 6**).

Second, the main characteristics differentiating responders and non-responders were defined and their presence in individual results were visually inspected and rated by two independent, blind raters. Following the direction of change found at 10Hz (alpha) and 22Hz (beta) for the responder and non-responder groups, individuals showing *a pattern of a meaningful decrease in alpha (~8-14Hz) and/or increase in beta (~18-30Hz) WPLI from week 1 to 12* were considered to fit the responder pattern, while patients showing *a pattern of a meaningful increase in alpha and/or decrease in beta WPLI from week 1 to 12* were considered to fit the non-responder pattern. Two authors (K.C. and G.W.) examined each individual outcome pattern and independently decided whether they conformed to the outlined definitions. Inter-rater reliability was quantified using Cohen’s Kappa: κ=0.76 for rating whether patients fit the responder pattern, and κ=0.57 for rating whether patients fit the non-responder pattern. Raters discussed discrepancies until consensus, which indicated that 39.5% of patients fit the pattern of their own group exclusively, 34.9% fit both patterns, 14.0% only showed the pattern of the opposite groups, and the remaining 11.6% did not conform to either pattern (7% did not reach significance).

**Figure 7:**
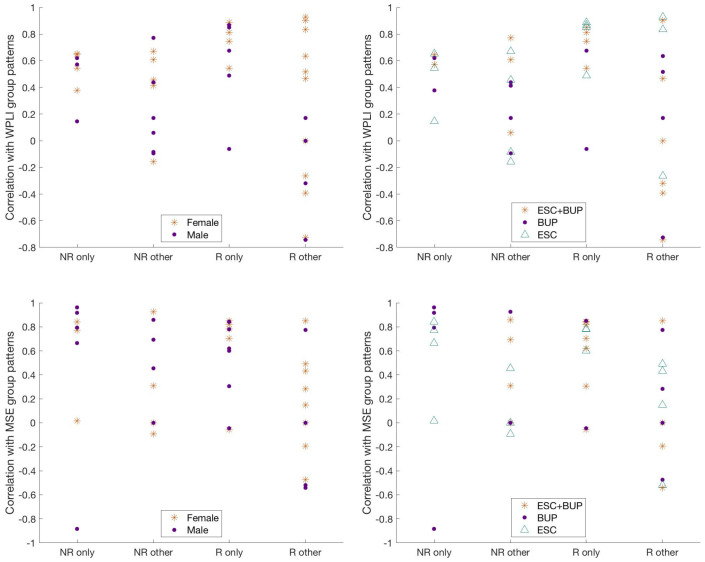
Correlation of individual WPLI (top row) and MSE (bottom row) patterns with participants’ own group patterns, grouped according to ratings based on visual inspection and color coded according to sex (left column) and treatment regiment (right column). NR only = individual non-responders whose patterns exclusively matched the non-responder group pattern; NR other = individual non-responders whose patterns either matched only the responder pattern, both the responder and non-responder patterns, or neither pattern; R only = individual responders whose patterns exclusively matched the responder group pattern; R other = individual responders whose patterns either matched only the non-responder group pattern, both the responder and non-responder patterns, or neither pattern; ESC+BUP, dual therapy with escitalopram and buproprion; BUP, monotherapy with buproprion; ESC, monotherapy with escitalopram. WPLI, weighted phase lag index; MSE, multiscale entropy.

**Table 2** shows the characteristics of patients who were rated as fitting their own WPLI pattern exclusively versus the other categories. Aside from individual responders who conformed to the responder group pattern showing higher correlations with their own WPLI group pattern compared to responders assigned to other categories (*p* = .005, did not survive Holm’s sequential Bonferroni test for multiple comparisons), there were no obvious clinical and demographic differences between individuals in the different responder and non-responder categories. We also illustrate the correlations between individual WPLI patterns and the WPLI pattern of participants’ own groups along the four categories in **Table 2**, colour coded according to treatment regimen and sex in **Figure 7** (top row). No clear patterns of individual variation in WPLI related to sex or treatment regimen emerged.

**Table 2:** Demographic and clinical characteristics (means ± standard error) of antidepressant treatment responders and non-responders divided based on which individuals matched the group WPLI patterns.

	Responders (N = 25)		Non-responders (N = 18)	
	*Responder pattern only (N = 10)*	*Other categories* *(N = 15)*	*Non-responder pattern only (N = 7)*	*Other categories* *(N = 11)*
*Sex (F/M)*	4/6	10/5	4/3	5/6
*Age*	38.0 ± 3.7 (range: 23-57)	32.5 ± 2.5 (range: 19-46)	40.3 ± 4.6 (range: 20-57)	47.6 ± 3.3 (range: 28-63)
*Education (years)*	16.7 ± 0.8*	14.5 ± 0.6	15.1 ± 0.8	17.1 ± 0.9
*Race/Ethnicity*	1 Asian; 9 White	2 Asian; 13 White	7 White	1 African; 10 White
*Comorbid anxiety (Yes/No)*	0/10	3/12	0/7	2/9
*Treatment regimen (ESC+BUP/BUP+placebo/ESC+placebo)*	4/2/4	8/4/3	2/2/3	3/4/4
*Baseline MADRS score*	29.4 ± 1.4	29.4 ± 1.3	33.1 ± 1.0	31.5 ± 1.7
*MADRS score at 1 week*	23.9 ± 2.8	22.6 ± 2.0	29.7 ± 2.6	26.7 ± 2.7
*MADRS score at 12 weeks*	5.1 ± 1.5	6.7 ± 1.3	25.4 ± 3.1	24.5 ± 2.5
*Correlation with own group WPLI pattern (>.4/<.4)*	9/1*	6/9	3/4	3/8
*Correlation with own group WPLI pattern*	0.67 ± 0.09**	0.13 ± 0.15	0.51 ± 0.07	0.30 ± 0.09
*Correlation with own group MSE pattern*	0.44 ± 0.16	0.26 ± 0.12	0.40 ± 0.15	0.40 ± 0.18

Group differences were examined using independent samples t-tests and Fisher’s exact test in Excel. **Significant differences between groups at p <.05 (with Holm’s sequential Bonferroni test to correct for multiple comparisons, as these were unplanned comparisons). *Group differences with p <.05 that did not survive the correction. F, female; M, male; ESC, escitalopram; BUP, bupropion; MADRS, Montgomery-Åsberg Depression Rating Scale; WPLI, weighted phase lag index; MSE, multiscale entropy.

### 3.5 Individual analyses - MSE

Non-rotated PLS was performed for all participants with two predefined contrasts: a linear contrast (1 0 -1) as the responder pattern (change across the three time points, top of **Figure 4**) and 1 1 -2 as the non-responder pattern (no change from baseline to week 1, change from weeks 1 to 12, top of **Figure 4**). While all individual analyses revealed significant results for both predefined LVs (all *p* <.001), 16% of patients did not show the same pattern of change over assessment sessions as defined in their own group contrast.

The similarity of the pattern of complexity across channels and time scales between the individual and group-level results was examined in two ways (see section 2.10 and **Figure 1D** for details). First, each individual’s complexity pattern was correlated with the complexity patterns identified in the group analyses. This procedure showed that 53.5% of individual outcome patterns correlated positively with moderate-high strength (*r* ≥ .4; 58) to that of their groups. Another 9.3% showed a weak (.1 < *r* < .4) positive correlation between their individual outcome and that of their group. Of the remaining individuals’ analyses, 11.6% yielded negative correlations, while 25.6% either showed negligible correlations (-.1 < *r* < .1) or did not show the predefined contrast and were therefore not correlated with group patterns (**Figure 6**).

Second, the main characteristics differentiating responders and non-responders were defined and their presence in individual results were visually inspected and rated by two independent, blind raters. Participants were characterized as fitting the responder pattern if they showed a *meaningful decrease in coarse scale MSE from baseline to week 1* in their first LV, and characterized as fitting the non-responder pattern if they demonstrated *no meaningful change in coarse scale MSE from baseline to week 1, and any change in coarse scale MSE from week 1 to week 12* in their second LV. Again, two raters (K.C. and G.W.) examined the significant patterns found in each individual analysis and independently decided whether they conformed to these definitions. Inter-rater reliability yielded κ=0.91 for rating whether patients fit the responder pattern, and κ=0.96 for ratings on whether patients fit the non-responder pattern. Based on consensus, 46.5% of patients exclusively showed the pattern of their own group, 25.6% fit both patterns, 14.0% exclusively showed the opposite pattern, and 14.0% showed neither. Patients in this last group, including 4 responders and 2 non-responders, showed an early *increase* in coarse scale complexity instead.

The characteristics of patients rated as conforming to their own group pattern exclusively versus those who did not are presented in **Table 3**. Again, the most notable difference was that responders exclusively showing the responder pattern exhibited higher correlations between their individual and group MSE patterns compared to responders assigned to the other categories; this comparison did not survive Holm’s sequential Bonferroni test to correct for multiple comparisons (*p* = .006). The correlations between individual MSE patterns and the MSE pattern of participants’ own groups along the four categories in **Table 3**, colour coded according to treatment regimen and sex are presented in **Figure 7** (bottom row). We observed no noticeable patterns of individual variation in MSE related to treatment regimen or sex.

**Table 3:** Demographic and clinical characteristics (means ± standard error) of antidepressant treatment responders and non-responders divided based on which individuals matched the group MSE patterns.

	Responders (N = 25)		Non-responders (N = 18)	
	*Responder pattern only (N = 12)*	*Other categories* *(N = 13)*	*Non-responder pattern only (N = 8)*	*Other categories* *(N = 10)*
*Sex (F/M)*	5/7	9/4	3/5	6/4
*Age*	30.6 ± 3.2 (range: 21-46)	38.5 ± 2.4 (range: 19-57)	46.5 ± 4.0 (range: 33-63)	43.4 ± 3.9 (range: 20-57)
*Education (years)*	15.8 ± 0.5	15.0 ± 0.8	16.0 ± 0.9	16.6 ± 0.9
*Race/Ethnicity*	1 Asian; 11 White	2 Asian; 11 White	1 African; 7 White	10 White
*Comorbid anxiety (Yes/No)*	1/11	2/13	1/7	1/9
*Treatment regimen (ESC+BUP/BUP+placebo/ESC+placebo)*	7/2/3	5/4/4	0/4/4	5/2/3
*Baseline MADRS score*	28.8 ± 1.4	29.9 ± 1.3	32.0 ± 1.5	32.3 ± 1.7
*MADRS score at 1 week*	20.3 ± 2.5	25.8 ± 1.9	27.5 ± 2.4	28.2 ± 2.9
*MADRS score at 12 weeks*	4.4 ± 1.1	7.5 ± 1.5	24.5 ± 2.4	25.2 ± 2.9
*Correlation with own group MSE pattern (>.4/<.4)*	9/3*	3/10	6/2	4/6
*Correlation with own group MSE pattern*	0.59 ± 0.10*	0.10 ± 0.13	0.51 ± 0.23	0.32 ± 0.12
*Correlation with own group WPLI pattern*	0.44 ± 0.15	0.26 ± 0.16	0.25 ± 0.12	0.48 ± 0.07

Group differences were examined using independent samples t-tests and Fisher’s exact test in Excel. * Group differences with p <.05 that did not survive the Holm’s sequential Bonferroni correction for multiple comparisons. F, female; M, male; ESC, escitalopram; BUP, bupropion; MADRS, Montgomery-Åsberg Depression Rating Scale; MSE, multiscale entropy; WPLI, weighted phase lag index.

## 4 Discussion

The current study identified group-based patterns of change in EEG connectivity and complexity that differentiated responders and non-responders. As a group, responders exhibited decreasing alpha connectivity, increasing beta connectivity, and widespread decreases in coarse scale complexity over the course of treatment. Nonresponders showed an opposite pattern of connectivity, and spatially limited decreases in coarse scale complexity. Single-participant analyses revealed that these differentiating group features only existed unambiguously in up to 61% of individuals. Others showed the pattern of the opposite group, both group patterns, or neither group pattern in their individual analysis. Therefore, although group analyses were able to detect neural characteristics of treatment success that apply to certain patients at an individual level, a substantial proportion of individuals is poorly represented.

For both EEG connectivity and complexity, our group findings were in line with some previous findings, but not others, as is generally the case in the depression literature. Namely, responders showed a decrease in alpha connectivity over treatment, most notably in left fronto-temporal and right occipito-parietal electrode pairs, similar to Iseger and colleagues (9) and Lee and colleagues (6). Contrarily, increased alpha connectivity has also been observed in response to antidepressant pharmacotherapy (8). Although others have also found weaker baseline connectivity in delta and theta bands to be associated with better response (6), we did not find pronounced group effects in these frequency ranges. In line with the work of Olbrich and colleagues (8), we observed beta connectivity increases with successful treatment in left central, parietal and frontal areas. In line with yet others’ work (16), we found that EEG complexity at lower temporal resolution (20-40ms) decreased with treatment, and this was prominent only in responders. This differs from findings highlighting a decrease in complexity at high temporal resolutions instead (16, 17), and would be worth examining in future studies.

Together, these variable group-level findings and the substantial individual variation found in our single-participant analyses provide a possible explanation as to why reliable EEG characteristics associated with antidepressant treatment response have not yet emerged. Depending on the EEG characteristic and how it was identified, 39-61% of our sample did not unambiguously show the same outcomes as their group, and a similar level of variability was observed in both responders and non-responders to treatment. These findings are consistent with the idea that multiple response patterns to antidepressant treatment exist (59). Taking alpha connectivity as an example, recent work shows that alpha connectivity profiles may differentially predict response to placebo versus antidepressant pharmacotherapy (sertraline; 60), highlighting the possibility that different alpha connectivity patterns may distinguish responders to different types of interventions. The decrease in alpha connectivity in treatment responders using group analyses in this and previous studies might thus only represent one of several response profiles that exist in patients with MD. Similarly, the variable findings in beta connectivity in our individual analyses suggest changes in beta connectivity are not consistent across all patients and might therefore explain the variable findings in previous literature on this frequency range.

The responder group pattern we report here involved a decrease in alpha connectivity and coarse scale complexity, and an increase in beta connectivity. Although increased alpha connectivity in patients with MD compared to controls has been interpreted in varying ways (9, 61), the decrease in alpha connectivity with successful treatment reported here supports the notion that this feature plays a key role in MD pathology and its treatment. The importance of this frequency band is further highlighted by frequent findings of altered alpha power and hemispheric asymmetry (e.g., 62, 63). Given that intra-hemispheric anterior-posterior beta connectivity has been associated with emotion regulation in neurotypical populations (64), increased beta over successful treatment could reflect increased top-down control over altered emotional processing in MD in response to treatment (65). Increased overall EEG complexity in MD has been linked to recruiting more neural resources when performing an emotion processing task than controls (13). Generally, increased signal complexity has been associated with a greater number of simultaneously activated systems (66). As MD has been associated with reduced ability to suppress default mode network activation and greater interconnectedness between affective and other information processing systems (67, 68), the decrease in complexity over successful treatment found here might indicate decreased dominance and interference by emotional processing circuits.

While our findings are in keeping with multiple response patterns existing within the population of patients with MD, they do not provide proof of this, as we only tested whether the patterns appearing at the group level were also present in individual patients. Indeed, our approach is markedly different from other studies aiming to address applicability of neuroimaging to individual patients. For example, clustering approaches aim to divide patients into functionally relevant MD subcategories, and machine learning methods aim to identify features that predict individual treatment outcomes. Although encouraging work has emerged (69–71), subtyping research has had limited success so far (59), and these studies are far from perfect: Most existing research relies on study samples that are too small and homogeneous to provide reliable results for clustering or machine learning approaches (72, 73). Additionally, findings from a recent machine learning study using a large multi-site dataset (N=1188) were not replicated, highlighting methodological challenges in analyzing high dimensional datasets using such approaches (59, 74, 75). Thus, examining the brain characteristics associated with antidepressant treatment response likely warrants the use of multiple, complementary approaches. We propose that the use of single-participant analyses could help advance this field by determining the degree of individual variation and capturing the range of patterns present at the individual level.

In the field of neuroimaging, select studies have already shown that stable individual-specific fMRI characteristics are associated with cognitive functioning and clinical symptoms (76–78). A recent preprint further indicates that individual characteristics of the salience network can be related to the development, presence and fluctuation of MD symptoms (79). Research on the use of EEG as a biometric to identify individuals suggests that similar stable individual-specific EEG characteristics exist (e.g., 80, 81). While we are unaware of studies examining individual EEG features in relation to cognition or mental health, it could be interesting to explore the range of individual patterns and their relation to MD symptoms and progression over time to see if they yield similar potential for clinical utility.

### 4.1 Limitations & future directions

Despite the novelty of the presented work, certain limitations exist. First, we grouped patients receiving different treatment regimens (i.e. escitalopram, bupropion or both) to ensure sufficient statistical power for our main analyses. Similarly, as the clinical trial was designed to compare different treatment arms, we were not able to differentiate between treatment and placebo effects, a noteworthy future direction. Our sample was also largely White, and from WEIRD (Western, Educated, Industrialized, Rich and Democratic; 82) societies, and thus findings cannot be generalized outside of such populations. While our sample was fairly balanced in terms of sex, we were also unable to test for sex differences due to power issues. In addition, our sample included patients with different MD symptom subtypes (e.g. melancholic, atypical) and several patients had comorbid anxiety disorders (given the high co-occurrence of anxiety in depressed individuals these individuals were not excluded). Although this heterogeneity in the sample complicates statistical analyses, it does give an accurate representation of the population seeking treatment for MD. At the same time, the treatment provided was not representative of what people with comorbid conditions may receive in clinical practice, as participants were not allowed to take psychotropic medications outside of those prescribed for the study. While heterogeneity may also have contributed to the individual variation we found in the individual analyses, we did not find any indication that individual variation in EEG connectivity and complexity patterns could simply be explained by differences in treatment regimen, sex, clinical profiles or other clinical and demographic characteristics (see **Tables 2**, **3**; **Figure 7**). That being said, future studies with larger samples should be conducted to explore the influence of heterogeneity of treatment and patient characteristics in more detail. They might also collect data during more narrow time windows during the day, or control for sleepiness/drowsiness in other ways (e.g., as a covariate in the analyses), which we were unable to do here. Similarly, it could be relevant to include more follow-up measurements across longer time periods, other types of individual information (e.g., neuroimaging) and outcome metrics (e.g., functional measures) in such studies, as well as explore alternative analysis methods (e.g., classification methods using Bayesian or machine learning approaches).

In addition, there are numerous ways to quantify similarity between individual and group patterns. The correlation procedure was objective, but also led to arbitrary limits for categorizing who did and did not match the group patterns (i.e. r ≥.4). The independent ratings avoided setting such arbitrary limits. While inter-rater reliability was high for ratings of MSE (κ = 0.91-0.96), there was less agreement for the ratings of WPLI (κ = 0.57-0.76), highlighting the subjectivity of this method. The lower inter-rater reliability for WPLI was likely due to the larger number of elements included in this analysis (378*99 compared to 28*20 for MSE), or could reflect more variety in WPLI response patterns compared to MSE patterns. Finally, we applied no correction for multiple comparisons in the individual analyses, as this would have made it more difficult to find the group patterns in individuals. However, lowering the significance threshold to α = .001 did not alter our MSE findings, and only changed 16% of the individual WPLI analyses (7 patients) from being significant to being insignificant. Overall, both measures of similarity showed that there was substantial individual variation in the connectivity and complexity group patterns.

## 5 Conclusion

Most existing work involving neural characteristics of antidepressant treatment success is based on responder/non-responder group differences. We show that substantial individual variation in EEG connectivity and complexity existed in a well-characterized sample of patients receiving pharmacotherapy for MD. Though speculative at this point, exploring the range of individual patterns and their relations to MD symptoms and stages in depth may lead to a better understanding of the heterogeneity in brain signals in MD. This also provides an alternative approach to identifying clinically relevant EEG features to group studies, which to date have not yielded convincing results. Regardless, future research should take individual variation into account when developing and considering the utility of EEG characteristics in informing clinical practice.

## Data availability statement

All EEG data analyzed in this manuscript are available in raw and preprocessed form on OSF (https://osf.io/f6pw3/), together with the data matrices that were prepared for the partial least squares analyses. The clinical and demographic data cannot be shared publicly because no ethics approval has been granted for sharing this data. Permission to access these data can be requested by contacting natalia.jaworska@theroyal.ca or njaworsk@uottawa.ca directly.

## Ethics statement

The studies involving humans were approved by Royal Ottawa Health Care Group and University of Ottawa Research Ethics Boards. The studies were conducted in accordance with the local legislation and institutional requirements. The participants provided their written informed consent to participate in this study.

## Author contributions

GW: Writing – review & editing, Writing – original draft, Visualization, Software, Methodology, Investigation, Formal analysis, Conceptualization. YE: Methodology, Investigation, Writing – review & editing, Formal analysis. KC: Writing – review & editing, Methodology, Investigation, Formal analysis. MS: Writing – review & editing, Methodology, Investigation, Formal analysis. PB: Writing – review & editing, Funding acquisition, Data curation. VK: Writing – review & editing, Funding acquisition, Data curation. NJ: Writing – review & editing, Project administration, Funding acquisition, Data curation, Conceptualization. AP: Writing – review & editing, Supervision, Resources, Methodology, Investigation, Funding acquisition, Conceptualization.
